# Single nucleotide polymorphisms in IFN-gamma, TNF-alpha, IL-6, IL-10, and TGF-beta in pulmonary and extrapulmonary tuberculosis in the State of Ceará, northeastern Brazil

**DOI:** 10.1590/0074-02760240147

**Published:** 2025-05-16

**Authors:** Roberta dos Santos Silva Luiz, Thales Alves Campelo, Caroliny Soares Silva, Lucas de Lima Nogueira, Soraya de Oliveira Sancho, Ana Karolliny Alves da Silva, Cristiane Cunha Frota, Filipe Anibal Carvalho-Costa

**Affiliations:** 1 Universidade Federal do Ceará, Faculdade de Medicina, Laboratório de Micobactérias, Fortaleza, CE, Brasil; 2 Fundação Oswaldo Cruz-Fiocruz, Instituto Oswaldo Cruz, Laboratório de Epidemiologia e Sistemática Molecular, Rio de Janeiro, RJ, Brasil

**Keywords:** tuberculosis, single nucleotide polymorphism, cytokines

## Abstract

**BACKGROUND:**

Single nucleotide polymorphisms (SNP) in genes encoding cytokines influence tuberculosis (TB) outcomes.

**OBJECTIVES:**

To characterise genotypes of the SNPs IFN-gamma +874 T > A, TNF-alpha -308 G > A, IL-6 -174 G > C, IL-10 -1082A > G, TGF-beta codon 10 T > C, and TGF-beta codon 25 G > C in patients with pulmonary (PTB) and extrapulmonary TB (EPTB).

**METHODS:**

82 PTB and 45 EPTB cases were compared, concerning genotype distribution of the mentioned SNPs, characterised via sequence-specific primer polymerase chain reaction (PCR).

**FINDINGS:**

Regarding IFN-gamma +874 T > A, AA genotype was the most frequent in both groups, TA was more frequent in PTB and TT in EPTB, with no statistical significance. For SNP TNF-alpha -308 G > A, GG was more frequent in both groups of patients. Regarding the IL-6 -174 G > C polymorphism, GG predominated in both groups, while CG and GG were significantly more frequent in patients with PTB and EPTB, respectively. Concerning IL-10 -1082 A > G, AA predominated in both PTB and EPTB. Concerning TGF-beta codon 10 T > C, CC predominated in PTB while TC predominated in EPTB, but the differences were not statistically significant. Genotype GG of TGF-beta codon 25 G > C predominated among PTB and EPTB patients.

**MAIN CONCLUSIONS:**

Except for IL-6, the genotype profile could not differentiate PTB and EPTB. Hence, the studied SNPs are not significantly associated with the extrapulmonary involvement of TB.

Tuberculosis (TB) is caused by *Mycobacterium tuberculosis*, an air-borne acid-fast bacillus transmitted via the inhalation of aerosolised particles exhaled by individuals with active respiratory disease.^1^ *M. tuberculosis* is an intracellular pathogen that infects phagocytic cells, more frequently affecting the lungs and causing pulmonary tuberculosis (PTB), but potentially spreading to other organs and causing extrapulmonary tuberculosis (EPTB).^2^ Miliary TB is a severe and multisystemic form of TB associated with the diffuse lymphohematogenous dissemination of *M. tuberculosis.* ^3^ In 2022, the World Health Organization (WHO) estimated approximately 7.5 million newly diagnosed TB cases, out of which 83% were PTB and 17% were EPTB.^4^ More than 74,385 new TB cases were registered in Brazil, including 582 cases of EPTB in 2022.^5^ Laboratory diagnosis of EPTB can be difficult, usually requiring a biopsy for confirmation.^6^ In addition, specific therapeutic regimens are needed for EPTB.^7^

Infection by *M. tuberculosis*, although potentially lethal and disabling, is not typically followed by symptomatic disease, with nearly 90% of individuals controlling bacillary multiplication, progressing to latent TB.^8^ This trend toward a balanced host-parasite relationship, liable to be lost if immunodeficiency is installed, may be explained by millenary co-evolution with a long-lasting human-*M. tuberculosis* interaction. This has been accompanied by the selective pressure of resistant human populations, capable of implementing an efficient immune response that can impair bacillus multiplication and the tissue damage it causes.^9^ ^,^ ^10^ Despite being a disease of poverty and having clear and strong social determinants, the burden of tuberculosis on a global scale is also influenced by genetic background.^11^

The immune response against TB begins with the recognition and phagocytosis of *M. tuberculosis* by alveolar macrophages and dendritic cells.^12^ It is well established that Th1 CD4 T cells expressing cytokines such as interferon-gamma (IFN-gamma) and tumour necrosis factor-alpha (TNF-alpha), among other pro-inflammatory mediators, play a major role in the immune response against *M. tuberculosis*. This occurs because Th1 cells activate phagocytes and enhance their anti-bacterial activity.^12^ Hence, the hypothesis that variation in genes encoding immune mediators - particularly those located in regions regulating gene expression and, consequently, levels of cytokine production - may represent risk factors for different forms of tuberculosis. In this context, specific genomic polymorphisms of the host have been related to the risk of developing TB^13^ and studies have assessed the influence of single nucleotide polymorphisms (SNPs) in genes encoding mediators of the immune response on TB outcomes.^14^ The characterisation of genetic polymorphisms involved in immune response in different forms of TB, therefore, could contribute to the characterisation of high-risk groups.

Among the studied immune mediators, IL6 is a pro-inflammatory cytokine that undergoes increased production in many human chronic inflammatory diseases. It is of critical importance for an effective immune response against *M. tuberculosis.* ^15^ Furthermore, SNPs in the IL-6 gene have been associated with a risk of symptomatic TB.^16^ Immune cells also produce regulatory cytokines such as interleukin - 10 (IL-10) and transforming growth factor-beta (TGF-beta), which negatively regulate the activation and proliferation of T lymphocytes and, consequently, the production of pro-inflammatory cytokines, leading to a Th1 immune response reduction.^17^ ^,^ ^18^ The balance of pro- and anti-inflammatory immune mediators in the context of Th1 and Th2 responses determines the contention of bacillary multiplication, the formation of granulomas, inflammation, tissue damage, and the resulting disease severity. The objective of this study is to assess and compare the frequency of well-recognised SNPs in genes encoding IFN-gamma, TNF-alpha, IL-6, IL-10, and TGF-beta in HIV-negative patients with PTB and EPTB attending two reference centres in northeastern Brazil.

## SUBJECTS AND METHODS

*Setting and study design* - This is a comparative case study, conducted in Fortaleza city, the capital city of the State of Ceará, in northeastern Brazil. Fortaleza has 2,428,678 inhabitants and is the fourth most populous Brazilian city. Participants were recruited from the outpatient clinics and wards of the São José Hospital for Infectious Diseases and the Walter Cantídio University Hospital. Both hospitals are reference tertiary centres for TB patients.

The study includes 127 individuals recently diagnosed with TB (PTB and EPTB), confirmed by culture or Gene Xpert MTB/RIF (a nucleic acid amplification test for simultaneous rapid tuberculosis diagnosis and rapid antibiotic sensitivity test), plus histopathological confirmation (in EPTB cases), following the criteria established by the Brazilian Ministry of Health.^19^ Patients with positive cultures for non-tuberculous mycobacteria, together with those found to have HIV co-infection, diabetes, liver disease, chronic kidney disease, or taking prolonged corticosteroid or immunosuppressive therapy, under antineoplastic chemotherapy and/or radiotherapy, and those under 15 years of age or over 80 years old, were excluded from the study. Two comparison groups were assembled: the PTB group with 82 patients and the EPTB group with 45 patients. In the EPTB group, patients with lymph node (n = 18), pleural (n = 13), and breast (n = 7) tuberculosis predominated; there were also patients diagnosed with tuberculosis affecting the osteoarticular system (n = 3), the central nervous system (n = 2), the ovary (n = 1) and the larynx (n = 1). Key patient information was obtained from laboratory and medical records.

*Characterisation of SNP in cytokine genes* - Genomic DNA from 10 mL of EDTA-anticoagulated blood was obtained with PureLink™ Genomic DNA Mini Kit (Thermo Fisher Scientific, Waltham, MA, USA). SNPs were detected via the sequence-specific primer polymerase chain reaction (PCR-SSP). A test assay for genotyping of IFN-gamma, TNF-alpha, IL-6, IL-10, and TGF-beta was utilised (Cytokine Genotyping Tray kit, One Lambda, Canoga Park, CA, EUA). The following SNPs were characterised: IFN-gamma +874 T > A, TNF-alpha -308 G > A, IL-6 -174 G > C, IL-10 -1082 A > G, TGF-beta codon 10 T > C, and TGF-beta codon 25 G > C.

*Statistical analysis* - For each polymorphic site of the different cytokines studied, the frequencies of each genotype in the different clinical phenotypes (EPTB and PTB) were compared by calculating odds ratios (ORs) (cross-product ratios) and their respective 95% confidence intervals (95% CIs). The significance of the associations was verified by Fisher’s exact test with a significance level set at p < 0.05. Allele frequencies were calculated considering, as numerator, how many times the allele is present (twice in homozygous genotypes and once in heterozygous genotypes) in each clinical phenotype and as denominator the total number of alleles in that polymorphic site. Allele frequencies were compared with Fisher’s exact test with a significance level set at p < 0.05. All data were analysed using SPSS software (IBM Corp. Released 2023. IBM SPSS Statistics for Windows, Version 29.0.2.0 Armonk, NY: IBM Corp.).

*Ethics* - Approved by the Research Ethics Committee of the Federal University of Ceará (Protocol No. CAAE23258013.2.0000.5044 and 545.314), the study has been conducted according to the Declaration of Helsinki principles. All participants signed the Free and Informed Consent Form and allowed samples to be collected.

## RESULTS

As presented in Table I, among the 82 PTB patients, 59 (72%) were male and 23 (28%) were female, while among the 45 EPTB cases, 19 (42%) were male and 26 (58%) were female (p < 0.001; chi-square test). The age range was 15-80 years for PTB and 17-70 years for EPTB. The mean age ± standard deviation among patients with PTB and EPTB were 38.4 ± 14.8 and 36.4 ± 15.5 (p = 0,708; Students’ t-test). The proportion of patients with rifampicin-resistant *M. tuberculosis*, as assessed with GeneXpert MTB/RIF, was 59/82 (72%) in the PTB group and 7/45 (15.6%) in the EPTB group (p < 0.001; chi-square test).

**TABLE I:** Distribution of pulmonary and extrapulmonary tuberculosis cases by sex, age and resistance to rifampicin

	PTB (n = 82)	EPTB (n = 45)^*^	p-value
Gender			
Male	59 (72%)	19 (42%)	< 0.001
Female	23 (28%)	26 (58%)	
Age			
Mean age ± standard deviation	38.4 ± 14.8	36.4 ± 15.5	0.708
Age range (years)	15-80	17-70	
Proportion with rifampicin-resistant *Mycobacterium tuberculosis* ^**^	59 (72%)	7 (15.6%)	< 0.001

^*^ Lymph node (n = 18); pleural (n = 13); breast (n = 7); osteoarticular system (n = 3); central nervous system (n = 2); ovary (n = 1); and larynx (n = 1). ^**^ Assessed with GeneXpert MTB/RIF. PTB: pulmonary tuberculosis; EPTB: extrapulmonary tuberculosis.

Among the patients studied, the number of those who could be genotyped in the PTB and EPTB groups varied for the different SNPs - as presented in Table II. The Table also demonstrates that, regarding the IFN-gamma +874 T > A polymorphism, the AA genotype was the most frequent in both the PTB and EPTB groups. The heterozygote TA genotype was more frequent in PTB than in EPTB, while the TT genotype was more frequent in EPTB, but neither difference was statistically significant. For the TNF-alpha -308 G > A polymorphism, the GG genotype was much more frequent in both groups of patients, followed by the heterozygote AG. Regarding the IL-6 -174 G > C polymorphism, although the GG genotype predominated in patients of both groups, CG and GG were significantly more frequent in patients with PTB and EPTB, respectively. The distribution of genotypes of the polymorphism IL-10 -1082 A > G was similar in both groups, with a predominance of AA in PTB and EPTB. Concerning the study of SNPs TGF-beta codon 10 T > C, CC predominated in PTB and TC in EPTB, however, differences were not statistically significant. Finally, genotype GG of TGF-beta codon 25 G > C predominated among PTB and EPTB patients.

**TABLE II:** Distribution of different genotypes and allele frequencies of polymorphic sites IFN-gamma +874 T > A, TNF-alpha -308 G > A, IL-6 -174 G > C, IL-10 -1082 A > G, TGF-beta codon 10 T > C, and TGF-beta codon 25 G > C in patients with pulmonary (PTB) and extrapulmonary tuberculosis (EPTB) attending two reference centres in Fortaleza city, State of Ceará, Brazil

	PTB	EPTB	OR (95% CI)	p*-*value
IFN-gamma +874 T > A	N = 65	N = 36		
AA (low^*^ )	26 (40%)	15 (41.7%)	0.93 (0.40 - 2.19)	1.000
TA (intermediate^*^ )	26 (40%)	8 (22.2%)	2.33 (0.92 - 5.90)	0.081
TT (high^*^ )	13 (20%)	13 (36.1%)	0.44 (0.17 - 1.10)	0.097
Allele A	78 (60%)	38 (52.8%)	1.34 (0.75 - 2.39)	0.373
Allele T	52 (40%)	34 (47.2%)	0.74 (0.41 - 1.33)	0.373
TNF-alpha -308 G > A	N = 68	N = 40		
GG (low^**^ )	53 (77.9%)	32 (80%)	0.88 (0.33 - 2.13)	1.000
AG (high^**^ )	15 (22.1%)	8 (20%)	1.13 (0.43 - 2.96)	1.000
Allele A	15 (11%)	8 (10%)	1.11 (0.45 - 2.76)	1.000
Allele G	121 (89%)	72 (90%)	0.89 (0.36 - 2.21)	1.000
IL-6 174G > C	N = 73	N = 40		
CC (low^***^ )	8 (11%)	5 (12.5%)	0.86 (0.26 - 2.83)	0.768
CG (intermediate^***^ )	29 (39.7%)	7 (17.5%)	3.10 (1.21 - 7.96)	0.019
GG (high^***^ )	36 (49.3%)	28 (70%)	0.41 (0.18 - 0.94)	0.046
Allele C	45 (30.8%)	17 (21.3%)	1.65 (0.87 - 3.13)	0.160
Allele G	101 (69.2%)	63 (78.7%)	0.60 (0.31 - 1.14)	0.160
IL-10 -1082 A > G	N = 29	N = 16		
AA	15 (51.7%)	11 (68.8%)	0.48 (0.13 - 1.75)	0.351
AG	10 (34.5%)	2 (12.5%)	3.68 (0.69 - 19.52)	0.164
GG	4 (13.8%)	3 (18.7%)	0.69 (0.13 - 3.57)	0.685
Allele A	40 (69%)	24 (75%)	0.74 (0.27 - 1.96)	0.631
Allele G	18 (31%)	8 (25%)	1.35 (0.50 - 3.57)	0.631
TGF-beta codon 10 (T > C)	N = 70	N = 36		
CC	30 (42.8%)	11 (30.6%)	1.70 (0.72 - 3.99)	0.292
TC	21 (30%)	16 (44.4%)	0.53 (0.23 - 1.13)	0.196
TT	19 (27.2%)	9 (25%)	1.11 (0.44 - 2.80)	1.000
Allele C	81 (57.9%)	38 (52.8%)	1.22 (0.69 - 2.17)	0.559
Allele T	59 (42.1%)	34 (47.2%)	0.81 (0.45 - 1.44)	0.559
TGF-beta codon 25 (G > C)	N = 70	N = 36		
CC	0 (0%)	4 (11.1%)	Undefined	
GC	10 (14.3%)	4 (11.1%)	1.33 (0.38 - 4.59)	0.768
GG	60 (85.7%)	28 (77.8%)	1.71 (0.61 - 4.81)	0.412
Allele C	10 (7.1%)	12 (33.3%)	0.38 (0.15 - 0.93)	0.054
Allele G	130 (92.9%)	60 (66.6%)	2.60 (1.06 - 6.35)	0.054

^*^ Genotypes associated with low, intermediate and high production of IFN-gamma;^20^ ^) **^ Genotypes associated with low and high production of TNF-alpha;^38^ ^) ***^ Genotypes associated with low, intermediate and high production of IL-6;^39^ ^)^ CI: confidence interval; OR: odd ratio.

By analysing the allele frequencies, the following distributions were observed: for IFN-gamma +874 T > A, a predominance of A in PTB and EPTB; for TNF-alpha -308 G > A, a substantial predominance of G in both groups; for IL-6 -174 G > C, a predominance of G in both groups; for IL-10 -1082 A > G, a predominance of A in both groups; for TGF-beta codon 10 T > C, a slight predominance of C in both PTB and EPTB; and for TGF-beta codon 25 G > C, a predominance of G in both groups, with a tendency for the frequency to be higher in PTB than in EPTB.

## DISCUSSION

By comparing PTB and EPTB, this study has searched for variations in genes encoding cytokines involved in the immune response against *M. tuberculosis.* Frequencies of different genotypes in the polymorphic sites IFN-gamma +874 T > A, TNF-alpha -308 G > A, IL-10 -1082 A > G, TGF-beta codon 10 T > C, and TGF-beta codon 25 G > C were similar in PTB and EPTB. The genotypes CG and GG of IL-6 -174 G > C were more frequent in PTB and EPTB, respectively.

Regarding the IFN-gamma +874 T > A polymorphism, patients from the present study with both PTB and EPTB had a high frequency of the A allele, both homozygous AA and heterozygous TA. These genotypes are associated with lower and intermediate expression, respectively, of the gene encoding IFN-gamma.^20^ The SNP is in the first intron of the IFN-gamma gene, the binding site for NFκB, the transcription factor driving this cytokine production. IFN-gamma is a key cytokine in the protective immune response against *M. tuberculosis*, being produced by CD4, CD8, and NK cells. IFN-gamma activates macrophages and stimulates a Th1 immune response, which is the most efficient against *M. tuberculosis*. Homozygous AA individuals have been demonstrated as producing lower levels of IFN-gamma.^20^ Plus, a meta-analysis concluded that, according to the studies published up to that time, the T allele of IFN-gamma +874 T > A polymorphism elicits a protective effect for symptomatic TB.^21^ In China, a case-control study assessing IFN-gamma +874 T > A polymorphism demonstrated the frequency of the A allele to be associated not only with TB but also with decreased Th1 cells percentage, as assessed by flow cytometry in peripheral blood.^22^ In Brazil, it has been demonstrated that the AA homozygous genotype presents an over two-fold risk of developing PTB.^23^ An Algerian study also found the IFN-gamma +874 AA genotype to be associated with a higher EPTB risk.^24^ Similarly, in a Kashmiri population, it was found an association of IFN-gamma +874 TT genotype with EPTB.^25^ In our study, although AA and TA predominated in both groups, a trend toward a higher frequency of TT in EPTB than in PTB was observed, but according to the available sample size without statistical significance.

In the present study, the genotyping of patients based on TNF-alpha -308 G > A polymorphism, found in the gene’s promoter region, revealed a much higher frequency of the GG genotype in both groups, followed by GA, with no significant differences in genotypes distribution between PTB and EPTB. TNF-alpha-mediated immune pathways are very important for containing TB. Produced by activated macrophages, T lymphocytes, and dendritic cells, TNF-alpha acts in synergy with IFN-gamma to improve the concentration of reactive nitrogen intermediates, recruiting immune cells and promoting the development of granulomas. This occurs within a Th1 immune response associated with infection control and the development of latent TB. Studies carried out to associate specific genotypes of the TNF-alpha -308 G > A polymorphism with TB outcomes have produced conflicting results. A meta-analysis suggested that, despite no specific TNF-alpha -308 G > A genotype representing a risk of symptomatic TB in the total population, a significant risk of the A allele was detected among Asians.^26^ In China, a study focused on patients with pneumoconiosis demonstrated the TNF-alpha -308 A allele to be associated with an increased risk of PTB.^27^ Another study, undertaken on patients with EPTB (osteoarticular TB) demonstrated an association of this clinical form with the GA genotype.^28^ Kumar et al.^29^ and Ates et al.,^30^ however, did not detect an association between the different genotypes of TNF-alpha -308 G > A and the occurrence of tuberculosis. Similarly, a meta-analysis performed by Pacheco et al., which aggregated data from ten studies, did not identify a significant association between specific TNF-alpha -308 G > A genotypes and tuberculosis development.^21^

Concerning the IL-6 -174 G > C polymorphic site characterisation, although GG was the most frequent genotype in both groups of patients, it was significantly more frequent in EPTB, whereas CG was significantly more frequent in PTB. IL-6 -174 G > C SNP is in the gene’s promoter region, while the GG genotype is associated with a higher gene expression. The importance of IL-6 in TB, along with its association with different outcomes, has been recognised. Plasmatic pre-treatment IL-6 concentration has been observed as a biomarker for unfavourable TB treatment outcomes.^31^ Macrophages, dendritic cells, and B cells produce IL-6. This is an important mediator of the acute phase response, stimulating B cell maturation, promoting Th2 differentiation, and inhibiting Th1 polarisation.^32^ A meta-analysis has demonstrated that IL-6 -174 G > C polymorphisms may confer susceptibility to TB.^33^ In Brazil, a decreased risk of PTB has been associated with IL6 -174C carriers (CC and CG).^16^ This result aligns with data from our study suggesting that most patients with PTB and EPTB have genotype GG, which is associated with high IL-6 production.

Unlike TNF-alpha and IFN-gamma, IL-10 can inhibit the synthesis of pro-inflammatory Th1 cytokines - synthesised by monocytes and, to a lesser extent, Th2 cells. In the present study, the characterisation of IL-10 -1082 A > G SNP was successful in only a subgroup of patients with PTB and EPTB due to technical difficulties, which hindered an accurate description of different genotype distributions. Among the patients who could be genotyped, the AA genotype was predominant among PTB cases. In the present study, from 29 patients with PTB and 16 with EPTB, genotype AA was present in more than one-half of both groups, with the A allele largely predominating over the G allele. The heterozygote genotype GA was associated with TB in a Kashmiri population.^25^ In Algeria, the allele A of SNP IL10 -1082 A > G, was associated with PTB, especially in its homozygous form (AA).^24^

TGF-beta is a cytokine that reaches high pulmonary concentration in TB^34^ ^,^ ^35^ with its suppressive effects on immune cells suggesting that its inhibition may improve TB outcome.^36^ Our data shows that a little more than half of the patients, with both PTB and EPTB, were characterised with genotypes at polymorphic sites [TGF-beta codon 10 T > C and codon 25 G > C] associated with high expression of the gene encoding TGF-beta. Recently, a significant association between SNPs up-regulating TGF-beta expression and tuberculosis susceptibility was demonstrated.^37^

Taken together, the data from the present study were not able to characterise most of the polymorphic sites studied, except for IL-6 - a specific genotype profile able to differentiate between PTB and EPTB. In addition, genotypes already recognised as associated with the development of symptomatic disease predominated in both patient groups. 

The data suggest that, after the initial infection, although there are polymorphisms associated with a greater risk of developing symptomatic disease, SNPs in cytokine genes associated with low or high production are, for the most part, not associated with extra-pulmonary TB involvement. Studies undertaken to enable a better understanding of the genetic factors associated with extrapulmonary tuberculosis should expand the number of loci evaluated and search for new polymorphisms. Taking a genomic perspective, such research should adopt population-based approaches featuring a larger number of patients, including latent tuberculosis, familial contacts, and different clinical forms.
